# A feasibility study using mid-upper arm circumference as the sole anthropometric criterion for admission and discharge in the outpatient treatment for severe acute malnutrition

**DOI:** 10.1186/s40795-021-00448-w

**Published:** 2021-08-12

**Authors:** Souna Garba, Halidou Salou, Fabienne Nackers, Amadou Ayouba, Montse Escruela, Ousmane Guindo, Mercé Rocaspana, Rebecca F. Grais, Sheila Isanaka

**Affiliations:** 1 Epicentre Niger, Maradi, Niger; 2 Médecins Sans Frontières Operational Center Brussels, Brussels, Belgium; 3 District Sanitaire de Madaoua, Madaoua, Niger; 4 Médecins Sans Frontières Operational Center Barcelona, Barcelona, Spain; 5 Epicentre, 14-34 avenue Jean Juarès, 75019 Paris, France

**Keywords:** Severe acute malnutrition, Wasting, Community-based management of acute malnutrition, Mid-upper arm circumference, Admission criteria, Discharge criteria, Niger

## Abstract

**Background:**

The World Health Organization recommends the use of a weight-for-height Z-score (WHZ) and/or mid-upper arm circumference (MUAC) as anthropometric criteria for the admission and discharge of young children for the community-based management of severe acute malnutrition. However, using MUAC as a single anthropometric criterion for admission and discharge in therapeutic nutritional programs may offer operational advantages to simplify admission processes at therapeutic nutritional centers and improve program coverage.

**Methods:**

This pragmatic, non-randomized, intervention study compared a standard outpatient nutritional program (*n* = 824) for the treatment of uncomplicated severe acute malnutrition using WHZ < − 3 and/or MUAC< 115 mm and/or bipedal edema for admission and discharge to a program (*n* = 1019) using MUAC as the sole anthropometric criterion for admission (MUAC< 120 mm) and discharge (MUAC ≥125 mm at two consecutive visits) in the Tahoua Region of Niger.

**Results:**

Compared to the standard program, the MUAC-only program discharged more children as recovered (70.1% vs. 51.6%; aOR 2.31, 95%CI 1.79–2.98) and fewer children as non-respondent or defaulters, based on respective program definitions. The risk of non-response was high in both programs. Three months post-discharge, children who were discharged after recovery in the MUAC-only program had lower WHZ and MUAC measures. Sixty-three children ineligible for the MUAC-only program but eligible for a standard program (MUAC ≥120 mm and WHZ < -3) were followed for twelve weeks and the anthropometric status of 69.8% of these children did not deteriorate (i.e. MUAC ≥120 mm) despite not immediately receiving treatment in the MUAC-only program.

**Conclusions:**

The results from this study share the first operational experience of using MUAC as sole anthropometric criterion for admission and discharge in Niger and overall support the consideration for MUAC-only programming: the MUAC-only model of care was associated with a higher recovery and a lower defaulter rate than the standard program with very few children found to be excluded from treatment with an admission criterion of MUAC < 120 mm. Further consideration of the appropriate MUAC-based discharge criterion as it relates to an increased risk of non-response and adverse post-discharge outcomes would be prudent.

## Background

It is estimated that at any given time at least 14 million children under the age of five years suffer from severe acute malnutrition (SAM), a condition that is associated with a significantly increased risk of morbidity and mortality [1]. In 2007, a United Nations joint declaration approved a new SAM management model combining outpatient treatment with ready-to-use therapeutic foods (RUTF) for cases without clinical complications and inpatient treatment for the stabilization of cases with clinical complications [2]. This model has proven to be both effective [3, 4] and cost-effective [5–7], and has allowed life-saving treatment to be offered to millions of children.

Since 2009, the World Health Organization (WHO) has recommended the use of a weight-for-height Z-score (WHZ) < − 3 and/or mid-upper arm circumference (MUAC) < 115 mm as anthropometric criteria for admission to therapeutic nutrition programs [8, 9]. Based on increasing experience with the community management of SAM and an aim to simplify treatment at therapeutic nutritional centers, the possibility of using MUAC as the sole anthropometric criterion for admission and discharge at therapeutic nutrition centers has been suggested. The arguments in favor of wider usage of MUAC are as follows: the simplicity of the measurements, low cost and the potential for increased coverage due to the ease and transparency of use and harmonization between screening and admission procedures.

The transition to a single MUAC threshold for admission to therapeutic management is complicated by the fact that MUAC and WHZ identify different children as being at risk for severe malnutrition [9–13], and operational experience using MUAC as the sole criterion for admission and discharge remains relatively limited. However, a recent randomized trial has implemented MUAC as the sole criterion for admission and discharge [14], and promising reports from programs based on MUAC in Burkina Faso and India suggest that good rates of recovery and weight gain can be achieved [15, 16]. Recent program data showing a close correlation between weight gain and MUAC during treatment for SAM also suggest that monitoring average MUAC, compared to weight gain, can be reliable and feasible [17, 18].

Based on the potential benefits and the feasibility of programs based on MUAC, and while waiting for approval at the national level of the use of MUAC as the sole anthropometric criterion for admission to treatment in Niger, Médecins Sans Frontières (MSF) – Barcelona Operational Center (OCBA) tested MUAC as the sole anthropometric criterion for admission and discharge for the therapeutic nutritional program for uncomplicated SAM in Madaoua, Niger in 2018–2019. We report the first operational experience with this model of care in Niger and the comparison of its results with a standard program using both WHZ and/or MUAC as the anthropometric criteria for admission and discharge [17, 18].

## Methods

### Study setting

Madaoua Health District in the Tahoua region is a rural area of south-central Niger where acute malnutrition is endemic with a seasonal peak from June until October. The Ministry of Public Health assures the community-based management of acute malnutrition in the district, including inpatient and outpatient feeding centers. Since 2006, MSF has provided support for inpatient and outpatient care in the Madaoua Health District, and at the time of the study, supported the two outpatient centers in Madaoua town and Sabon Guida. In 2016, the two outpatient centers admitted more than 5000 children for the treatment of SAM based on either the presence ofWHZ <− 3 and/or a MUAC < 115 mm and/or bipedal edema.

### Study design

This was a pragmatic, non-randomized intervention study in two sites (Madaoua and Sabon Guida) to compare the operational experience of an outpatient treatment program using MUAC as the sole anthropometric criterion for admission and discharge for uncomplicated SAM (MUAC-only) to that using WHZ and/or MUAC as the combined anthropometric criteria (standard program). All children presenting to the two study sites were evaluated for eligibility for study enrollment.

### Intervention

The standard program as per the national protocol of Niger was provided at one outpatient health center (Madaoua) [19]. At the health facility, all children aged 6–59 months presenting with a MUAC < 115 mm and/or a WHZ < − 3 and/or bipedal edema were eligible for SAM treatment and received all systematic treatments (e.g. deworming, vaccination, malaria treatment in case of positive rapid test, systematic amoxicillin) and a weekly ration of RUTF based on weight as per the national protocol. Children were followed on a weekly basis at the outpatient center for a minimum of three weeks and maximum of 8 weeks. Children were referred for inpatient care in case of medical complications. A child was considered to have recovered in absence of edema and medical complications and both WHZ ≥ − 2 and MUAC ≥125 mm at 2 consecutive visits. At the second outpatient health center (Sabon Guida), MUAC was introduced as the sole anthropometric criterion for admission (MUAC < 120 mm and/or bipedal edema) and discharge (MUAC ≥125 mm at two consecutive visits). The more inclusive threshold at admission of MUAC < 120 mm compared to the standard program using MUAC < 115 mm was chosen to increase the sensitivity of using a sole criterion for admission based on MUAC only compared to the combined use of WHZ < − 3 and/or MUAC < 115 mm [11]. In addition to weekly facility-based follow-up during treatment, all children at both sites were followed at home at three months (± one week) after program discharge to record anthropometric status, history of SAM relapse (as reported by the caregiver) and any visit to a health care professional. Study enrolment at the health centers lasted for 12 months (June 2018 – June 2019) to allow for seasonal variability. All clinical care and follow-up were identical between sites and as per the national protocol.

Finally, recognizing that MUAC can identify different children as having SAM compared to WHZ [9–13], we aimed to also understand the anthropometric evolution of children who would have been eligible for treatment under a standard program (e.g. admission defined by WHZ and/or MUAC and/or bipedal edema) but not eligible under a new MUAC-only program. Therefore, children with MUAC ≥120 and WHZ < − 3 (and no edema) were enrolled for at-home follow-up to measure their nutritional and medical status in the absence of immediate treatment at 4, 8, and 12 weeks following their presentation to the outpatient center.

### Statistical analysis

Clinical and nutritional status at the time of admission and during follow-up, as well as program outcomes at discharge, were recorded in routine patient files. Program outcomes included nutritional recovery (defined as the absence of edema and medical complications and both WHZ ≥ − 2 and MUAC ≥125 mm at 2 consecutive visits in Madaoua; and as the absence of edema and medical complications and MUAC ≥125 mm at 2 consecutive visits in Sabon Guida), transfer to another outpatient center, default (defined as an absence at two consecutive weekly visits), non-response (defined as not achieving nutritional recovery in the respective program by 8 weeks) and death. The study further calculated duration of treatment and response to nutritional treatment among recovered children (i.e. average daily weight and MUAC gain) and nutritional status up to three months after discharge among children discharged from the program. Baseline characteristics and program outcomes were compared between the MUAC-only and the standard program using logistic regression to estimate odds ratios (OR) with 95% confidence interval (95% CI) for binary outcomes or linear regression to estimate mean differences with 95% CI for continuous variables. Analyses were stratified by MUAC upon admission (< 115 mm; ≥ 115 mm) and adjusted for age, sex, HAZ and clinical variables imbalanced between the participants upon admission at the *p* <  0.05 significance level. In secondary analysis, models were further adjusted for MUAC and WHZ upon admission as well. All *p* values were two-sided, with p <  0.05 considered as statistically significant. Data analyses were performed using STATA (version 15; College Station, TX, USA).

### Complementary qualitative approach

To gain a richer understanding of the unexpectedly high risk of default and non-response observed during data collection, qualitative interviews were conducted among 55 caregivers in the catchment area of Sabon Guida (21 caregivers of non-responders in 4 villages) and Madaoua (19 caregivers of non-responders and 15 caregivers of defaulters in 6 villages). Caregivers were randomly selected from a list of non-responders and defaulters. All provided verbal consent for participation and were interviewed at home. In addition, interviews were also conducted with four therapeutic food re-sellers. Two study members trained in qualitative research methods carried out the individual interviews in the local language using semi-structured open questionnaires until saturation was reached. Written notes taken during the interviews were analyzed with thematic coding.

## Results

### Children admitted to the two outpatient program sites

A total of 1843 children were enrolled in the study between June 2018 to June 2019: 1019 in Sabon Guida with the MUAC-only program and 824 in Madaoua with the standard program. The age and sex of admitted children was similar between sites, but the children differed in anthropometric status and morbidities present at admission (Table 1). As expected given the differing admission criteria, a WHZ < -3 was more frequent in Madaoua while MUAC 115–119 mm was more frequent in Sabon Guida. Stunting was more common in Sabon Guida, whereas clinical morbidities were more frequent in Madaoua.

**Table 1:** Child characteristics upon admission at the outpatient centers of Sabon Guida and Madaoua, Tahoua Region, Niger, June 2018–June 2019

Characteristics	Sabon Guida MUAC-only program (***n*** = 1019)	Madaoua Standard program (***n*** = 824)	***p***
Age (mean ± standard deviation [SD])	16.9 (7.9)	16.8 (7.5)	0.711
6–11 months, n (%)	279 (27.4)	226 (27.4)	0.995
12–23 months, n (%)	471 (46.2)	379 (46.0)	
≥ 24 months, n (%)	269 (26.4)	219 (26.6)	
Female sex, n (%)	536 (52.6)	421 (51.1)	0.519
Weight, kg (mean ± SD)	6.21 ± 1.02	6.34 ± 1.07	0.006
Height, cm (mean ± SD)	69.4 ± 5.7	71.0 ± 6.2	< 0.001
WHZ (mean ± SD)	−3.25 ± 0.92	−3.49 ± 0.72	< 0.001
WHZ < -3, n (%)	609 (59.8)	667 (81.0)	< 0.001
MUAC, mm (median; IQR)	115 [110; 117]	113 [110; 116]	0.003
< 115, n (%)	492 (48.3)	543 (65.9)	
115 to < 120 mm, n (%)	510 (50.1)	173 (21.0)	
≥ 120 mm, n (%)	17 (1.6)	108 (13.1)	< 0.001
Edema, n (%)	23 (2.3)	25 (3.0)	0.298
Height for age z-score [HAZ] (mean ± SD)	−3.52 ± 1.35	− 2.93 ± 1.48	< 0.001
HAZ < -2, n (%)	893 (87.6)	598 (72.6)	< 0.001
New admission, n (%)	990 (97.3)	795 (96.5)	0.343
Re-admission/relapse, n (%)	29 (2.8)	29 (3.5)	
Morbidity upon admission			
Diarrhea, n (%)	357 (35.0)	438 (53.2)	< 0.001
Vomiting, n (%)	26 (2.6)	117 (14.2)	< 0.001
Fever, n (%)	108 (10.6)	361 (43.8)	< 0.001
Cough, n (%)	114 (11.2)	335 (40.7)	< 0.001
Running nose, n (%)	22 (2.2)	76 (9.2)	< 0.001
Dehydration (moderate/severe), n(%)	26 (2.6)	58 (7.0)	< 0.001
Positive malaria rapid test, n (%)	167 (16.4) 167(16.39%)	96 (11.7)	< 0.001

### Program outcomes

Compared to the standard program, we found higher recovery (70.1% in Sabon Guida vs. 51.6% in Madaoua; aOR 2.31, 95% CI: 1.79–2.98) and lower rates of default and non-response associated with the MUAC-only program (Table 2). The risk of death did not differ by site. Similar trends in program outcomes between sites were seen among children with MUAC upon admission < 115 mm and ≥ 115 mm, though program outcomes tended to be better among children with MUAC ≥115 mm upon admission.

**Table 2:** Program outcomes at the outpatient centers of Sabon Guida and Madaoua, Tahoua Region, Niger, June 2018–June 2019

	Sabon Guida MUAC-only program (*n* = 1019)	Madaoua Standard program (*n* = 824)						
	n (%)	n (%)	Crude OR (95%CI)	*p*	Adjusted^a^ OR (95%CI)	*p*	Adjusted^b^ OR (95%CI)	*p*
**All children**								
**Type of discharge**	**1019**	**824**						
Recovery	714 (70.1)	425 (51.6)	2.20 (1.81–2.66)	< 0.001	2.31 (1.79; 2.98)	< 0.001	1.68 (1.28; 2.22)	< 0.001
Default	42 (4.1)	78 (9.5)	0.41 (0.28–0.61)	< 0.001	0.28 (0.17; 0.45)	< 0.001	0.36 (0.22;- 0.60)	< 0.001
Death	21 (2.1)	15 (1.8)	1.13 (0.58–2.22)	0.711	1.44 (0.61; 3.44)	0.401	2.07 (0.84; 5.11)	0.112
Non-response	231 (22.7)	294 (35.7)	0.53 (0.43–0.65)	< 0.001	0.57 (0.44; 0.75)	< 0.001	0.75 (0.56; 0.99)	0.047
Transfer (to another outpatient center)	11 (1.1)	12 (1.5)	0.74 (0.32–1.68)	0.470	0.48 (0.16; 1.42)	0.189	0.53 (0.17; 1.65)	0.279
**Children with MUAC < 115 mm upon admission**								
**Type of discharge**	**492**	**543**						
Recovery	272 (55.3)	234 (43.1)	1.63 (1.27; 2.08)	< 0.001	1.58 (1.14; 2.19)	0.005	1.60 (1.16; 2.23)	0.004
Default	25 (5.1)	60 (11.1)	0.43 (0.26; 0.69)	< 0.001	0.29 (0.17; 0.54)	< 0.001	0.29 (0.16; 0.53)	< 0.001
Death	14 (2.9)	10 (1.8)	1.56 (0.68; 3.54)	0.288	1.49 (0.54; 4.10)	0.434	1.48 (0.53; 4.06)	0.446
Non-response	172 (35.0)	232 (42.7)	0.72 (0.56; 0.92)	0.011	0.90 (0.64; 1.25)	0.548	0.89 (0.64 1.25)	0.531
Transfer (to another outpatient center)	9 (1.8)	7 (1.3)	1.42 (0.52; 3.86)	0.484	0.66 (1.94; 2.28)	0.519	0.66 (0.19; 2.29)	0.520
**Children with MUAC ≥ 115 mm upon admission**								
**Type of discharge**	**527**	**281**						
Recovery	442 (83.9)	191 (67.9)	2.45 (1.74; 3.44)	< 0.001	2.57 (1.59; 4.15)	< 0.001	1.84 (1.05; 3.22)	0.032
Default	17 (3.2)	18 (6.4)	0.48 (0.24; 0.96)	0.038	0.32 (0.13; 0.81)	0.015	0.60 (0.20; 1.88)	0.385
Death	7 (1.3)	5 (1.7)	0.74 (0.23; 2.36)	0.615	2.64 (0.45; 15.59)	0.282	5.16 (0.45; 58.57)	0.185
Non-response	59 (11.2)	62 (22.1)	0.44 (0.30; 0.65)	< 0.001	0.40 (0.23; 0.70)	0.001	0.51 (0.27; 0.95)	0.036
Transfer (to another outpatient center)	2 (0.4)	5 (1.8)	0.21 (0.04–1.09)	0.063	0.24 (0.01; 3.36)	0.290	0.01 (0.00; 3.711)	0.133
**Duration and response to treatment among recovered children**			**Crude mean difference (95%CI)**	***p***	**Adjusted**^a^**mean difference (95%CI)**	***p***	**Adjusted**^a^**mean difference (95%CI)**	***p***
**All children**	**714**	**425**						
Duration of treatment (days)	43.3	45.7	−2.5 (−4.2; − 0.7)	0.006	−2.9 (−5.3; − 0.6)	0.012	− 1.6 (− 4.0; 0.7)	0.181
Weight gain (g/kg/day)	3.6	4.8	−1.2 (− 1.5; − 1.0)	< 0.001	−1.2 (− 1.6; − 0.8)	< 0.001	− 0.8 (− 1.2; − 0.4)	< 0.001
MUAC gain (mm/day)	0.29	0.31	−0.02 (− 0.03; − 0.001)	0.038	− 0.01 (− 0.036; 0.01)	0.203	− 0.01 (− 0.03; 0.01)	0.506
**Children with MUAC < 115 mm upon admission**	**272**	**234**						
Duration of treatment (days)	48.9	48.9	−0.002 (− 2.4; 2.4)	0.999	− 1.2 (− 4.3; 2.0)	0.468	−1.3 (− 4.5; 1.8)	0.416
Weight gain (g/kg/day)	4.1	4.8	− 0.7 (−1.1; − 0.3)	< 0.001	− 0.5 (− 1.0; − 0.03)	0.039	−0.6 (− 1.1; − 0.1)	0.015
MUAC gain (mm/day)	0.36	0.36	0.002 (−0.02; 0.02)	0.829	0.01 (−0.02; 0.04)	0.584	0.01 (−0.02; 0.04)	0.599
**Children with MUAC ≥ 115 mm upon admission**	**442**	**191**						
Duration of treatment (days)	39.8	41.8	− 2.0 (− 4.4; 0.4)	0.096	−1.2 (− 4.5; 2.1)	0.463	−3.6 (−7.5; 0.30)	0.070
Weight gain (g/kg/day)	3.2	4.8	−1.6 (− 2.0; − 1.2)	< 0.001	−1.6 (− 2.2; − 1.1)	< 0.001	−1.1 (− 1.8; −0.5)	< 0.001
MUAC gain (mm/day)	0.25	0.25	−0.001 (− 0.02; 0.02)	0.900	0.00 (− 0.02; 0.035)	0.712	− 0.03 (− 0.06; 0.001)	0.057

^a^Adjusted for age, sex, HAZ, and morbidities on admission

^b^Additionally adjusted for MUAC and WHZ on admission

At discharge, most non-responders (200/231, 86.6% in Sabon Guida and 290/294, 98.6% in Madaoua; *p* = 0.010) still suffered from SAM following 8 weeks of treatment (based on either MUAC< 115 mm and/or WHZ < -3 and/or presence of bipedal edema) of which 172/231 (74.5%) in Sabon Guida and 232/294 (78.9%) in Madaoua had a MUAC < 115 mm (*p* = 0.229) following 8 weeks of treatment. Among children who recovered, the average duration of treatment was higher in the standard program (Madaoua) overall. Daily weight gain was higher in the standard program (Madaoua) compared to the MUAC-only program (Sabon Guida), but there was no difference in MUAC gain between sites.

According to the study protocol, children discharged from the nutritional program (e.g. recovered or did not respond, *n* = 945 in Sabon Guida and *n* = 719 in Madaoua) were followed-up three months after discharge (Table 3) and > 90% of children were located 3-months post discharge. Among the children recovered, mortality up to 3-months post discharge was low in both groups (n deaths = 6 in Sabon Guida, n deaths =0 in Madaoua). Three months post discharge, children discharged from the MUAC-only program had lower WHZ and MUAC measures and had more often received care for a new episode of malnutrition (reported by the caregiver) or had more often visited a health professional for medical reasons.

**Table 3:** Nutritional and clinical status at the home visit three months after discharge from the outpatient centers of Sabon Guida and Madaoua, Tahoua Region, Niger, June 2018–June 2019

	Recovered			Non-responders		
	Sabon Guida MUAC-only program (***n*** = 714)	Madaoua Standard program (***n*** = 425)		Sabon Guida MUAC-only program (***n*** = 231)	Madaoua Standard program (***n*** = 294)	
	n (%)	n (%)	***p***	n (%)	n (%)	***p***
Wrong address	29 (4.1)	23 (5.4)	0.463	5 (2.1)	8 (2.7)	0.324
Child absent	14 (2.0)	6 (1.4)		13 (5.6)	9 (3.1)	
*Death*	*6 (0.8)*	*0 (0.0)*		*10 (4.3)*	*5 (1.7)*	
*Travel/migration*	*7 (1.0)*	*6 (1.4)*		*3 (1.3)*	*3 (1.0)*	
*Hospitalization*	*1 (0.1)*	*0 (0.0)*		*0 (0.0)*	*1 (0.3)*	
Child present	671 (94.0)	396 (93.2)		213 (92.2)	277 (94.2)	
**Among children present**	**(*n*= 671)**	**(*n*= 396)**		**(*n*= 213)**	**(*n*= 277)**	
Weight, kg (mean ± SD)	7.78 ± 1.24	8.29 ± 1.26	< 0.001	6.71 ± 1.10	7.02 ± 1.16	0.002
Height, cm (mean ± SD)	73.0 ± 5.1	74.9 ± 5.6	< 0.001	69.5 ± 4.5	70.9 ± 5.0	0.001
WHZ (mean ± SD)	−1.68 ± 0.95	−1.46 ± 0.88	< 0.001	−2.41 ± 1.18	−2.26 ± 1.06	0.141
WHZ < -3, n (%)	60 (8.9)	23 (5.8)	0.065	64 (30.1)	61 (22.0)	0.043
HAZ (mean ± SD)	−3.04 ± 1.30	−2.56 ± 1.42	< 0.001	−3.37 ± 1.48	−3.02 ± 1.53	0.012
HAZ < -2	526 (78.4)	253 (63.9)	< 0.001	173 (81.2)	207 (74.7)	0.088
MUAC (median; IQR)	127 [123; 134]	130 [125; 135]	< 0.001	119 [112; 125]	120 [115; 126]	0.013
< 115 mm	37 (5.5)	7 (1.7)	0.002	63 (29.6)	59 (21.3)	0.123
115–119 mm	59 (8.8)	22 (5.6)		44 (20.7)	52 (18.8)	
120–125 mm	114 (17.0)	60 (15.1)		48 (22.5)	76 (27.4)	
> 125 mm	461 (68.7)	307 (77.5)		58 (27.2)	90 (32.5)	
Edema, n (%)	0 (0.0)	0 (0.0)	–	5 (2.4)	1 (0.4)	0.047
SAM^a^	74 (11.0)	25 (6.3)	0.010	85 (39.9)	86 (31.1)	0.119
*Receiving nutritional treatment at the time of the visit*	*17*	*1*		*18*	*12*	
*Discharged from nutritional treatment after re-admission*	*5*	*2*		*7*	*6*	
*No nutrition treatment prior the visit*	*52*	*22*		*60*	*68*	
MAM^a^	142 (21.2)	72 (18.2)		72 (33.8)	104 (37.6)	
*Receiving nutritional treatment at the time of the visit*	*26*	*5*		*9*	*8*	
*Discharged from nutritional treatment after re-admission*	*2*	*1*		*6*	*5*	
*No nutrition treatment prior the visit*	*114*	*66*		*57*	*91*	
Not SAM or MAM	455 (67.8)	299 (75.5)		56 (26.3)	87 (31.4)	
*Receiving nutritional treatment at the time of the visit*	*9*	*3*		*3*	*4*	
*Discharged from nutritional treatment after re-admission*	*7*	*2*		*2*	*1*	
*No nutrition treatment prior the visit*	*439*	*294*		*51*	*82*	
Treatment in a nutritional program, n (%)	66 (9.8)	14 (3.5)	< 0.001	45 (21.1)	36 (13.0)	0.016
Location						
*Inpatient center, n (%)*	*5 (7.7)*	*3 (21.4)*		*7 (15.6)*	*5 (13.9)*	
*Outpatient center, n (%)*	*52 (80.0)*	*8 (57.1)*		*36 (80.0)*	*30 (83.3)*	
*Supplementary feeding program, n (%)*	*8 (12.3)*	*3 (21.4)*		*2 (4.4)*	*1 (2.8)*	
*Month of admission to care*						
*1st month*	*16 (24.2)*	*1 (7.1)*		*17 (37.8)*	*6 (16.7)*	
*2nd month*	*24 (36.4)*	*4 (28.6)*		*16 (35.6)*	*12 (33.3)*	
*3rd month*	*26 (39.4)*	*9 (64.3)*		*12 (26.7)*	*18 (50.0)*	
*Status of nutritional treatment:*						
*Ongoing at the time of the visit*	*52 (78.8)*	*9 (64.3)*		*30 (66.7)*	*24 (66.7)*	
*Recovered*	*8 (12.1)*	*3 (21.4)*		*6 (13.3)*	*4 (11.1)*	
*Default*	*0 (0.0)*	*0 (0.0)*		*0 (0.0)*	*1 (2.8)*	
*Transfer*	*4 (6.1)*	*2 (14.3)*		*6 (13.3)*	*5 (13.9)*	
*Non response*	*2 (3.0)*	*0 (0.0)*		*3 (6.7)*	*2 (5.6)*	
Reason if visiting health service, n (%)	150 (22.4)	21 (5.3)	< 0.001	45 (21.1)	22 (7.9)	< 0.001
*Fever, n (%)*	*56 (38.6)*	*7 (35.0)*		*16 (36.4)*	*6 (28.6)*	
*Diarrhea, n (%)*	*45 (31.0)*	*8 (40.0)*		*16 (36.4)*	*7 (33.3)*	
*Cough, n (%)*	*18 (12.4)*	*2 (10.0)*		*7 (15.9)*	*4 (19.1)*	
*Difficulty breathing, n (%)*	*7 (4.8)*	*0 (0.0)*		*0 (0.0)*	*1 (4.7)*	
*Others, n (%)*	*19 (13.1)*	*3 (15.0)*		*5 (11.4)*	*3 (14.3)*	

^a^According to the national protocol, based on MUAC (SAM < 115 mm; MAM 115 to < 125 mm) and/or WHZ (SAM < −3; MAM < −2) and/or edema measured at the time of the visit

### Children ineligible in the MUAC-only program

Over a period of 12 months, we identified 63 “MUAC-only ineligible children” (MUAC ≥120 mm and WHZ < − 3) at Sabon Guida, representing 6% of the 1019 children admitted at this site. At the end of the twelve week-follow-up, 44 (69.8%) remained ineligible for treatment under the MUAC-only program (e.g MUAC ≥120 mm) (Table 4). Sixteen (25.4%) children became eligible for treatment (e.g. MUAC < 120 mm and/or edema), most before 4 weeks and were admitted to the nutritional program (1 inpatient care and 15 outpatient care). One child died and two were lost to follow-up due to family migration.

**Table 4:** Characteristics of the “MUAC-only ineligible children” upon admission and during 12 weeks’ follow-up, Sabon Guida, Tahoua Region, Niger, June 2018–June 2019

Characteristics	Sabon Guida MUAC-only program (*n* = 63)
Age (mean ± SD)	22.5 (10.8)
6–11 months, n (%)	7 (11.2)
12–23 months, n (%)	28 (44.4)
≥ 24 months, n (%)	28 (44.4)
Female sex, n (%)	22 (34.9)
Weight, kg (mean ± SD)	7.2 ± 1.1
Height, cm (mean ± SD)	75.3 ± 6.7
WHZ (mean ± SD)	−3.40 ± 0.37
MUAC, mm (median; IQR)	121 [120; 124]
120 to ≤125 mm	53 (84.1)
> 125 mm	10 (15.9)
HAZ (mean ± SD)	−2.98 ± 1.24
HAZ < -2, n (%)	48 (76.2)
Outcome after 12 weeks	
Admitted in the nutritional program	16 (25.4)
Death	1 (1.6)
Loss to follow-up due to migration	2 (3.2)
Remained MUAC-only ineligible	44 (69.8)

### Complementary qualitative approach

To gain a better understanding of the unexpectedly high risk of default and non-response observed during the study, additional qualitative interviews were conducted with caregivers. Caregivers of non-responders from both Madaoua and Sabon Guida reported barriers to accessing care that included financial constraints, geographical difficulties, limited caregiver time (especially during the rainy season), and insecurity. Most perceived a positive improvement in their child’s health during treatment at the outpatient therapeutic feeding center. Numerous caregivers of non-responders reported having thought their child had been discharged cured from the nutritional program and were unaware that the child had been considered to have not responded to treatment at 8 weeks.

Therapeutic food was consistently reported to be managed at home by the caregiver, rarely by another household member. The majority of interviewed caregivers of children who had not responded reported to have shared their therapeutic food rations with other children living in the same or the neighboring household. Though caregivers reported acknowledging the therapeutic foods to be a treatment, they suggested sharing was unavoidable due to social pressure and to other children seeing and asking for the therapeutic food as well. Sharing appeared more frequent in Sabon Guida than in Madaoua. In Madaoua, the re-sale of therapeutic foods was more frequent likely due to the more accessible re-sale market network in Madaoua town. Such re-sale was described as a necessary practice to earn money for the household and reported by some women to involve only the unused therapeutic foods. Re-sellers of therapeutic foods confirmed the practice of re-sale in the local market, suggesting a common occurrence would be for caregivers to sell all or a few sachets of therapeutic food upon leaving the health center each week. The sachets were sold for 100 to 125 CFA each (0.18 to 0.23 USD) and re-sold in the market for 150 CFA (0.27 USD), which would raise money for use on other market goods and/or to pay for transport home.

Defaulters were investigated in Madaoua, where caregivers reported a number of barriers to reach the outpatient health center including illness or death in the family, travel (up to 2 h and longer during the rainy season), lack of motivation due to slow progress of their child’s recovery, shortage of therapeutic foods at the health center at the last visit, and difficulty managing the household stock of therapeutic foods given the social pressure to share. Some caregivers reported not returning to the health center due to shame related to their child’s condition and perceived prejudices from others thinking that they are unable to feed their family. Some caregivers finally reported feeling uncomfortable with health center staff who threatened the child would be removed from treatment if their anthropometric status did not improve. Nearly all mothers suggested that financial support for transport to the health center and continued education/sensitization would facilitate sustained attendance at the health centers.

## Discussion

This study compared a standard nutritional program for the treatment of uncomplicated SAM using WHZ and/or MUAC for admission and discharge to a MUAC-only program where MUAC was used as the sole anthropometric criterion for admission and discharge. The MUAC-only program used a MUAC threshold of < 120 mm, higher than the standard threshold < 115 mm, for admission in order to increase the sensitivity of the MUAC-only threshold to offer treatment to children with a MUAC between 115 and < 120 mm who may be potentially at increased risk of morbidity and mortality. This operational experience with a MUAC-only model of care found overall higher recovery and a lower defaulter rate than in the standard program.

In this setting, the use of different anthropometric admission criteria resulted in admitting children of different nutritional profiles in the two outpatient therapeutic feeding centers, consistent with previous reports [10–13]. Children included in the MUAC-only program were shorter and more often stunted but had fewer morbidities on admission. Consistent with the admission criteria in the MUAC-only program, children included in Sabon Guida had a higher mean MUAC and WHZ, with substantial proportion of children enrolled with moderate acute malnutrition (> 25% with MUAC between 115 and 119 mm and a WHZ between − 2 and ≥ − 3 on admission) and would not have been included in a standard program. It may be expected that the more favorable anthropometric profile of children included in the MUAC-only program would contribute to improved nutritional recovery, as seen elsewhere [15, 18, 20]. While we did observe greater recovery in the MUAC-only program compared to the standard program (70.1% vs. 51.6%), the difference in recovery remained after statistical adjustment for MUAC and WHZ on admission. Differential recovery may therefore be due to differences in the ease of reaching the respective definitions of recovery that differed by site (e.g. MUAC ≥125 mm at 2 consecutive visits in the MUAC-only program vs MUAC ≥125 mm and WHZ ≥ − 2 at 2 consecutive visits in the standard program), but further research is required.

Overall, the risk of non-response was high in this study. One-fifth of the children in the MUAC-only program and one-third of the children in the standard program did not meet the discharge criteria from their respective centers after having completed eight weeks of treatment. The high burden of non-response should be of particular concern as the majority of these children were SAM, frequently with a MUAC < 115 mm, despite being treated for 8 weeks. Qualitative results suggest that non-response was not well understood by caregivers, indicating that clear, adapted and positive communication should be ensured by the health staff during treatment and at the time of discharge. Further consideration of the reasons for high non-response in this setting, including the appropriate discharge threshold within a MUAC-only program, is warranted.

The risk of death and default was below the international recommendations [21], but the risk of default was notably twice as high in the standard program than in the MUAC-only program. Qualitative interviews among caregivers in the standard program support a variety of barriers to accessing care (e.g. financial constraints, geography, insecurity) but also highlight areas for program improvement (e.g. improved staff communication and relationships with caregivers), which may have varied by site.

Among the children who recovered and were admitted with MUAC ≥115 mm, the average duration of treatment tended to be shorter in the MUAC-only program. This result may be associated with the discharge criteria of the MUAC-only program not additionally requiring WHZ ≥ − 2 during two consecutive visits. The average daily weight gain (g/kg/day) was higher in the standard program than in the MUAC-only program, which may be expected given the more severe WHZ on admission [15, 18] .

In previous analysis, we reported on program outcomes achieved using a MUAC-based anthropometric discharge in Burkina Faso [15]. That previous study showed overall favorable program outcomes using MUAC ≥124 mm as the sole anthropometric criterion for discharge compared to proportional weight gain, but did not include post discharge follow-up. In the present study that included follow up three months after post discharge, we found that re-admission was more frequently observed in the MUAC-only program compared to the standard program where WHZ and/or MUAC were used as the anthropometric criteria for discharge. This may be in part attributed to the fact that readmission was based on the broader eligibility criteria of a MUAC < 120 mm, which would have (re-)admitted children with moderate acute malnutrition not otherwise eligible for (re-)admission in a standard program. Three months post discharge, children in the MUAC-only program had lower WHZ and MUAC compared to the standard program, which might suggest a weaker nutritional recovery in the MUAC-only program. Appropriate post-discharge care to support sustained recovery remains an important area where additional evidence is needed to inform effective interventions.

While increasing the eligibility threshold of MUAC on admission from 115 to 120 mm was intended to increase the sensitivity of the admission criterion, we anticipated that a certain number of children would be deemed newly ineligible for treatment under the MUAC-only program, as MUAC and WHZ are known to identify different children. Contrary to what has been reported elsewhere [22, 23], we found a very small number of children to be excluded from treatment using a MUAC-only model with admission defined by MUAC < 120 mm and the absence of bipedal edema (*n* = 63 ineligible compared to 1019 children admitted at the same site). After 12 weeks of at-home follow-up, the majority of these children (69.8%) did not deteriorate (i.e. MUAC ≥120 mm) despite not immediately receiving treatment in the MUAC-only program.

## Conclusions

This study shares the first comparative operational experience of using MUAC as sole anthropometric criterion for admission and discharge from an outpatient nutritional program in Niger. While characteristics of the children differed at the two sites due to the non-randomized design, results overall support further consideration of MUAC-only programming, with a higher recovery rate and lower non-response and defaulter rates than in the standard program admitting children using WHZ and/or MUAC as anthropometric criteria. MUAC-only nutritional treatment programs may provide an operational advantage to facilitate screening and enrolment and increase access to treatment, however, further consideration of the appropriate MUAC-based discharge criterion as it relates to potential adverse post-discharge outcomes would be prudent.

## Data Availability

The deidentified dataset supporting this research can be made available from the corresponding author, following a reasonable submitted request.

## Abbreviations

SAM: Severe acute malnutrition
RUTF: Ready-to-use therapeutic food
WHO: World Health Organization
WHZ: Weight-for-height z score
MUAC: Mid-upper-arm circumference
MSF: Médecins sans Frontières
HAZ: Height-for-age z score
OR: Odds ratio
MAM: Moderate acute malnutrition

## References

[1] UNICEF WHO (2016). The World Bank: Joint child malnutrition estimates - Levels and trends.

[2] WHO, WFP, UNSCN, UNICEF: Joint Statement on Community-Based Management of Severe Acute Malnutrition. In.: Available at: http://www.who.int/maternal_child_adolescent/documents/a91065/en/. (Accessed December 2014). 2007.

[3] Ciliberto MA, Sandige H, Ndekha MJ, Ashorn P, Briend A, Ciliberto HM, Manary MJ (2005). Comparison of home-based therapy with ready-to-use therapeutic food with standard therapy in the treatment of malnourished Malawian children: a controlled, clinical effectiveness trial. Am J Clin Nutr.

[4] Manary MJ, Ndkeha MJ, Ashorn P, Maleta K, Briend A (2004). Home based therapy for severe malnutrition with ready-to-use food. Arch Dis Child.

[5] Bachmann MO (2010). Cost-effectiveness of community-based treatment of severe acute malnutrition in children. Expert Rev Pharmacoecon Outcomes Res.

[6] Puett C, Sadler K, Alderman H, Coates J, Fiedler JL, Myatt M (2013). Cost-effectiveness of the community-based management of severe acute malnutrition by community health workers in southern Bangladesh. Health Policy Plan.

[7] Wilford R, Golden K, Walker DG (2012). Cost-effectiveness of community-based management of acute malnutrition in Malawi. Health Policy Plan.

[8] World Health Organization: Guideline updates on the management of severe acute malnutrition in infants and children. In.: Available at: http://www.who.int/nutrition/publications/guidelines/updates_management_SAM_infantandchildren/en/. (Accessed December 2014). 2013. 24649519

[9] World Health Organization (2009). UNICEF: WHO child growth standards and the identification of severe acute malnutrition in infants and children.

[10] Bern C, Nathanail L (1995). Is mid-upper-arm circumference a useful tool for screening in emergency settings?. Lancet.

[11] Luque Fernández M, Delchevalerie P, Van Herp M. Accuracy of MUAC in the detection of severe wasting with the new WHO growth standards. Pediatrics. 2010;126(1):e195-201. 10.1542/peds.2009-2175. Epub 2010 Jun 29. 10.1542/peds.2009-2175 20587675

[12] Isanaka S, Guesdon B, Labar A, Hanson K, Langendorf C, Grais R (2015). Comparison of clinical characteristics and treatment outcomes of children selected for treatment of severe acute malnutrition using mid upper arm circumference and/or weight-for-height Z-score. PLoS One.

[13] Laillou A, Prak S, de Groot R, Whitney S, Conkle J, Horton L, Un SO, Dijkhuizen MA, Wieringa FT (2014). Optimal screening of children with acute malnutrition requires a change in current WHO guidelines as MUAC and WHZ identify different patient groups. PLoS One.

[14] Bailey J, Opondo C, Lelijveld N, Marron B, Onyo P, Musyoki EN, Adongo SW, Manary M, Briend A, Kerac M (2020). A simplified, combined protocol versus standard treatment for acute malnutrition in children 6-59 months (ComPAS trial): a cluster-randomized controlled non-inferiority trial in Kenya and South Sudan. PLoS Med.

[15] Isanaka S, Hanson K, Frison S, Andersen C, Cohuet S, Grais R (2019). MUAC as the sole discharge criterion from community-based management of severe acute malnutrition in Burkina Faso. Matern Child Nutr.

[16] Burza S, Mahajan R, Marino E, Sunyoto T, Shandilya C, Tabrez M, Kumari K, Mathew P, Jha A, Salse N, Mishra KN (2015). Community-based management of severe acute malnutrition in India: new evidence from Bihar. Am J Clin Nutr.

[17] Binns P, Dale N, Hoq M, Banda C, Myatt M (2015). Relationship between mid upper arm circumference and weight changes in children aged 6–59 months. Arch Public Health.

[18] Goossens S, Bekele Y, Yun O, Harczi G, Ouannes M, Shepherd S (2012). Mid-upper arm circumference based nutrition programming: evidence for a new approach in regions with high burden of acute malnutrition. PLoS One.

[19] Ministère de la Santé du Niger, UNICEF, OMS: Protocole national de prise en charge intégrée de malnutrition aigue. 2016.

[20] Binns PJ, Dale NM, Banda T, Banda C, Shaba B, Myatt M (2016). Safety and practicability of using mid-upper arm circumference as a discharge criterion in community based management of severe acute malnutrition in children aged 6 to 59 months programmes. Arch Public Health.

[21] Ouyang H, Vanrooyen M, Gruskin S (2009). The sphere project: next steps in moving toward a rights-based approach to humanitarian assistance. Prehosp Disaster Med.

[22] Ahn E, Ouma C, Loha M, Dibaba A, Dyment W, Kim J, Beck N, Park T (2020). Do we need to reconsider the CMAM admission and discharge criteria? An analysis of CMAM data in South Sudan. BMC Public Health.

[23] Guesdon B, Couture A, Pantchova D, Bilukha O (2020). Do we need to reconsider the CMAM admission and discharge criteria?; an analysis of CMAM data in South Sudan. BMC Nutr.

